# Supplementary material for: Altered PBP4 and GdpP functions synergistically mediate MRSA-like high-level, broad-spectrum β-lactam resistance in Staphylococcus aureus
Source: mBio. 2024 Mar 26;15(5):e02889-23. doi: 10.1128/mbio.02889-23 (PMC11077961; doi:10.1128/mbio.02889-23)
Supplement: Supplemental data — Supplemental tables and figures. [file mbio.02889-23-s0002.docx]

**Altered PBP4 and GdpP functions synergistically mediate MRSA-like high-level, broad-spectrum β-lactam resistance in *Staphylococcus aureus***

Li-Yin Lai^1,2&^, Nidhi Satishkumar^1,2&^, Sasha Cardozo^1,2^, Vijay Hemmadi^1,2^, Leonor B. Marques^3^, Liusheng Huang^5^, Sergio R. Filipe^3,4^, Mariana G. Pinho^3^, Henry F. Chambers^6^ and Som S. Chatterjee^1,2#^

^1^Department of Microbial Pathogenesis, School of Dentistry, University of Maryland Baltimore, USA

^2^Institute of Marine and Environmental Technology (IMET), Baltimore, USA

^3^Instituto de Tecnologia Química e Biológica António Xavier, Universidade Nova de Lisboa, Oeiras, Portugal

^4^UCIBIO-REQUIMTE, Departamento de Ciências da Vida, Faculdade de Ciências e Tecnologia, Universidade Nova de Lisboa, Caparica, Portugal

^5^Drug Research Unit, Department of Clinical Pharmacy, University of California, San Francisco, San Francisco, CA, USA

^6^Division of Infectious Diseases, School of Medicine, University of California San Francisco, San Francisco, CA, USA

^&^Equal contributors

**Running Head: MRSA-like non-classical β-lactam resistance mechanism**

^#^Corresponding author:

Som S. Chatterjee

Columbus Center

701 Pratt Street

Baltimore, MD 21202

Tel: 410-234-8890. E-mail: [schatterjee@umaryland.edu](mailto:schatterjee@umaryland.edu)

**Keywords:** *gdpP*, *pbp4*, β-lactam resistance, and Methicillin-Resistant Lacking *mec* (MRLM)

**Supplementary Legends**

**Supplementary Tables**

**Table S1. List of bacterial strains used in this study.**

**Table S2. List of primers used in this study.**

**Supplementary Figures**

**Figure S1: Classification of mutations seen in laboratory-generated strains.**

**Figure S2: Schematic representation of classification of MRLM strains from previous studies presented in Figure 1.**

**Figure S3: Loading control for fig 2D**. Following imaging to detect Bocillin binding to PBPs, the gel presented in **Fig 2D** was stained with Coomassie Brilliant Blue R-250, destained (40% methanol, 10% acetic acid) and imaged using the Azure 600 imager as a loading control.

**Figure S4: Growth assay for strains to determine NGB-resistance over a period of time (A)** Without NGBs **(B)** With 2 mg/L Nafcillin **(C)** With 4 mg/L Oxacillin. Deletion of *gdpP* resulted in a growth defect when cultured without NGBs. In presence of NGBs, only Wt and Wtex P*pbp4** *pbp4*** Δ*gdpP* survived.

**Figure S5: Effect of the deletion of *gdpP* on antibiotic tolerance by TD test.** Elaborated results of TD-test analysis represented in **Fig 6**. Images shown for step 1 were taken after treatment with **(A)** Nafcillin and **(B)** Oxacillin for 18 hours. Images for step 2 were taken 2 days after treatment with glucose disks. The 2^nd^ row is a magnified image of the disk from the 1^st^ row for each step. While there were no colonies present after glucose exposure for Wtex and Wtex P*pbp4** *pbp4*** strains, they did appear for the strains that contained Δ*gdpP*. Step I shows the inhibition zone on the first day after the bacterial lawn was cultured with a 6 mm disk containing 1 µg antibiotics. There is a clear inhibition zone surrounding the disk. Step II shows the tolerant colonies inside the inhibition zone after replacing the antibiotics disk with a 4 mg glucose disk and culturing for another 2 days. **(C)** The number of tolerant colonies were measured in the inner 50% inhibition zone as demonstarted.

**Figure S6: Hemolysis assay.** Overnight cultures of bacterial strains were spotted onto TSA-blood plates (5% sheep blood) in increasing volumes (2 µL, 5 µL, 10 µL) as indicated, and were incubated at 37°C overnight, following which the plate was stored to 4°C before recording the hemolysis pattern.

**Figure S7. Schematic representation of the potential mechanisms in which PBP4 and CDA mediate NGB resistance due to alterations in *pbp4* and *gdpP*.** (A) Normal regulation and functioning of PBP4 leads to it first being membrane-bound, following which it performs peptidoglycan cross-linking. Functional GdpP leads to cleavage of CDA, thus maintaining cellular homeostasis. (B) Regulatory-site and missense mutations associated with PBP4 leads to over-expression of a structurally altered protein, that leads to increased peptidoglycan cross-linking. A truncated GdpP, due to mutations associated with the DHH/DHHA1 domain is unable to cleave CDA, causing it to accumulate within the cell. In presene of NGBs, this CDA could either interact directly or indirectly with PBP4 to alter its localization or stability, or could play a peptidoglycan synthesis. Finally, CDA could also have an unindentified interacting partner that plays a role in mediating synergistic NGB resistance.

**Supplemental file 1: Mutations in *pbp4* and *gdpP* detected in clinical strains**  (provided as a separate excel file)

| **Table S1. List of bacterial strains used in this study.** | | | |
| --- | --- | --- | --- |
| **Strains** | **Description** | | **Reference** |
| Wt | | Clinical MRSA isolate, SF8300, that belongs to the USA300 background strain | (1) |
| Wtex | | SF8300ex*; mecA* and *blaZ* excised SF8300 strain | (1) |
| Wtex Δ*gdpP* | | *gdpP* deleted SF8300ex strain | (2), This study |
| Wtex *Ppbp4** *pbp4*** | | 36-bp duplication at 290 bp upstream  of *pbp4* start codon; amino acid substation in PBP4 at E^183^A and F^241^R | (3), This study |
| Wtex *Ppbp4** *pbp4*** Δ*gdpP* | | *gdpP* deleted SF8300ex *Ppbp4** *pbp4*** | This study |
| Wtex *Ppbp4** *pbp4*** S^75^A | | SF8300ex *Ppbp4** *pbp4*** carries an amino acid substation in PBP4 at S^75^A | (2), This study |
| Wtex *Ppbp4** *pbp4*** S^75^A Δ*gdpP* | | *gdpP* deleted SF8300ex *Ppbp4** *pbp4*** S^75^A | This study |
| Wtex P*pbp4** *pbp4*** [E] | | SF8300ex P*pbp4** *pbp4*** carrying pTxΔ16 | This study |
| Wtex P*pbp4** *pbp4*** Δ*gdpP* [E] | | SF8300ex P*pbp4** *pbp4*** Δ*gdpP* [*gdpP*]  carrying pTxΔ16 | This study |
| Wtex P*pbp4** *pbp4*** Δ*gdpP* [*gdpP*] | | SF8300ex P*pbp4** *pbp4*** Δ*gdpP* [*gdpP*]  carrying pTxΔ [*gdpP*] | This study |
| RN4220 | | Laboratory *S. aureus* strain | BEI Resources |
| *E. coli* DH5α | |  | Invitrogen |

| **Table S2. List of primers used in this study.** | |  | |
| --- | --- | --- | --- |
| **Primer** | **Sequence (5`-3`)** | | **Purpose** |
| SC-3 | TTTGATATCTTGAGTCAATTACGTG | | *gdpP* sequencing |
| SC-4 | TTAATTGGGCACGATAACCAACAC | | *gdpP* sequencing |
| SC-5 | ACTGGAATATCAACCAACAGAACAACG | | *gdpP* sequencing |
| SC-6 | TACATCACTGTTGATTCAAGACGTG | | *gdpP* sequencing |
| MecA-presence-for | TTCTAAAAGCGATAATGGTGAAGTAGAAATGACTGAACGTCC | | *mecA* determination |
| MecA-presence-rev | ATGATATAAACCACCCAATTTGTCTGCCAGTTTCTCCTTGTTTC | | *mecA* determination |
| pbp4-for | TTTCCGTAAGTAGGAATAACCATTGC | | *pbp4* sequencing |
| pbp4-rev | AAATATAAAACGGACAAGTTTCGCAGC | | *pbp4* sequencing |
| pbp1-for | TTCTTATGAACGCATATACGAAAAGG | | *pbp1* sequencing |
| gdpP-P1 | GGGGACAAGTTTGTACAAAAAAGCAGGCTTGTTAATTTTCATTAAAGAGGTTAAAATAATAGCTATAGTTAAAAATATGG | | *gdpP* knockout |
| gdpP-P2 | TATTCCACCTCTATTCACTTTTTAGAATTATTTTTCATGATTCG | | *gdpP* knockout |
| gdpP-P3 | AAGTGAATAGAGGTGGAATAATGAAAGTAATTTTTACACAAGATGTTAAAGGTAAAGGTAAAAAAGG | | *gdpP* knockout |
| gdpP-P4 | GGGGACCACTTTGTACAAGAAAGCTGGGTCTTCAGCTGTTTCATACACTTGTCCTAAGACGTCTCGAATGTCTTTAAAGC | | *gdpP* knockout |
| gdpP-upstrm-1 | TGTTAATTTTCATTAAAGAGGTTAAAATAATAG | | *gdpP* up and downstream sequencing |
| gdpP-upstrm-2 | TCGCTTGTCTAAACCCTTCTTCTAATATTTGG | | *gdpP* up and downstream sequencing |
| gdpP-upstrm-3 | ATGCGAATATGACCCAAATATTAGAAGAAG | | *gdpP* up and downstream sequencing |
| gdpP-upstrm-4 | TATTCCACCTCTATTCACTTTTTAGAATTATTTTTC | | *gdpP* up and downstream sequencing |
| gdpP-Dnstrm-1 | ATGAAAGTAATTTTTACACAAGATGTTAAAG | | *gdpP* up and downstream sequencing |
| gdpP-Dnstrm-2 | TTAACAGGTACATTCGTATATCCTAGGGAATG | | *gdpP* up and downstream sequencing |
| gdpP-Dnstrm-3 | ATTCATTCCCTAGGATATACGAATGTACCTG | | *gdpP* up and downstream sequencing |
| gdpP-Dnstrm-4 | CTTCAGCTGTTTCATACACTTGTCCTAAGAC | | *gdpP* up and downstream sequencing |
| pbp4-upstrm-1 | AGTTTGCAATTTCAGATTGTGTAC | | *pbp4* up and downstream sequencing |
| pbp4-upstrm-2 | AACAGAAGAGGTAAAACTATGAAACGAG | | *pbp4* up and downstream sequencing |
| pbp4-upstrm-3 | TACCTCTTCTGTTTGAAATTTATAGTTAATTC | | *pbp4* up and downstream sequencing |
| pbp4-upstrm-4 | GAATTGGAAAAGGGAAGATTAACGCTTT | | *pbp4* up and downstream sequencing |
| pbp4-dnstrm-1 | AACATACTAAAAACGGACAAGTTGCAC | | *pbp4* up and downstream sequencing |
| pbp4-dnstrm-2 | TAGAGAAATATACGAATTGTGGCGACAAAC | | *pbp4* up and downstream sequencing |
| pbp4-dnstrm-3 | TTCGTATATTTCTCTATCCCAATTCTCACC | | *pbp4* up and downstream sequencing |
| pbp4-dnstrm-4 | GAAGATTTTAATAGATATATCACAG | | *pbp4* up and downstream sequencing |
| pbp4-1 | ATGAAAAATTTAATATCTATTATCATC | | *pbp4* sequencing |
| pbp4-2 | TACTTTGTTGGTGCAAATGTACGTAATCTTG | | *pbp4* sequencing |
| pbp4-3 | TTGCACCAACAAAGTATAAAGACCAAGAAC | | *pbp4* sequencing |
| pbp4-4 | TTATTTTCTTTTTCTAAATAAACGATTGATTATC | | *pbp4* sequencing |
| PBP4-P3-new | AAAAGGGAAGATTAACGCTTTAACATACTAAAAACGGACAAGTTGCACATTATAAAGCTGCGAAACTTGTCCGTTTTATATTTATTTTATAATAACC | | *pbp4* knockout |
| PBP4-P4-new | GGGGACCACTTTGTACAAGAAAGCTGGGTGAAGATTTTAATAGATATATCACAGAAATTATGAAAATAAGACAACGAGTCATGGAAATG | | *pbp4* knockout |
| gdpP del-mut scrn for | TACTTATAAAACATGAAAGTTATTATTC | | *gdpP* knockout |
| gdpP del-mut scrn rev | TTGGTCTAAATCTCGATATCCTGTAG | | *gdpP* knockout |
| Ppbp4* (CRB)-pImay-Not1-for | AGTTTGCGGCCGCAGATTGTGTACTTGTCGATATCTTTTG | | *Ppbp4** (CRB) mutation in SF8300ex |
| Ppbp4* (CRB)-rev | ACAAAAAATGCAATAGAAATATTCTATCATATAAATGTTATGAGCGGTATTTTG | | *Ppbp4** (CRB) mutation in SF8300ex |
| Ppbp4* (CRB)-for | ATATTTCTATTGCATTTTTTGTATTTATATGATAGAATATTTCTATTGC | | *Ppbp4** (CRB) mutation in SF8300ex |
| Ppbp4* (CRB)-pImay-kpn1-rev | TCTTGGTACCTTGTTGGTGCAAATGTACGTAATCTTG | | *Ppbp4** (CRB) mutation in SF8300ex |
| Ppbp4* (CRB) verification | ACAAAAAATGCAATAGAAATATTCTATCATATAAATACAAAAAATGC | | *Ppbp4** (CRB) mutation verification |
| pJB38-for | TTCCCCGAAAAGTGCCACCTGACGTCTAAG | | pJB38 cloning determination |
| pJB38-rev | AGCGAAAATGCCTCACATTTGTGCCACC | | pJB38 cloning determination |
| pbp4-pJB38-Kpn1-for | AGAGGTACCATAATTAAAACGAATATCGGTATCGTTATAAATG | | creation of *pbp4*** with pJB38 |
| pbp4-pJB38-Sal1-rev | AGTGTCGACAATGTTACTATTACCTGAACAAAGGC | | creation of *pbp4*** with pJB38 |
| pbp4(S75A)-for | AAGTGGAATCCAGCGGCTATGACTAAATTAATGACAATG | | *pbp4* (S^75^A) mutation |
| pbp4(S75A)-rev | TTAATTTAGTCATAGCCGCTGGATTCCACTTAGTATCG | | *pbp4* (S^75^A) mutation |
| GFP_BamHI_FP | AGGATCCTAAAAAGTGAATAGAGGTGGAATAATGTCAAAAGGAGAAGAATTATTTACAG | | Cloning GFP into pTxΔ |
| GFP_MluI_RP | ATACGCGTTTACTTATATAATTCATCC | | Cloning GFP into pTxΔ |


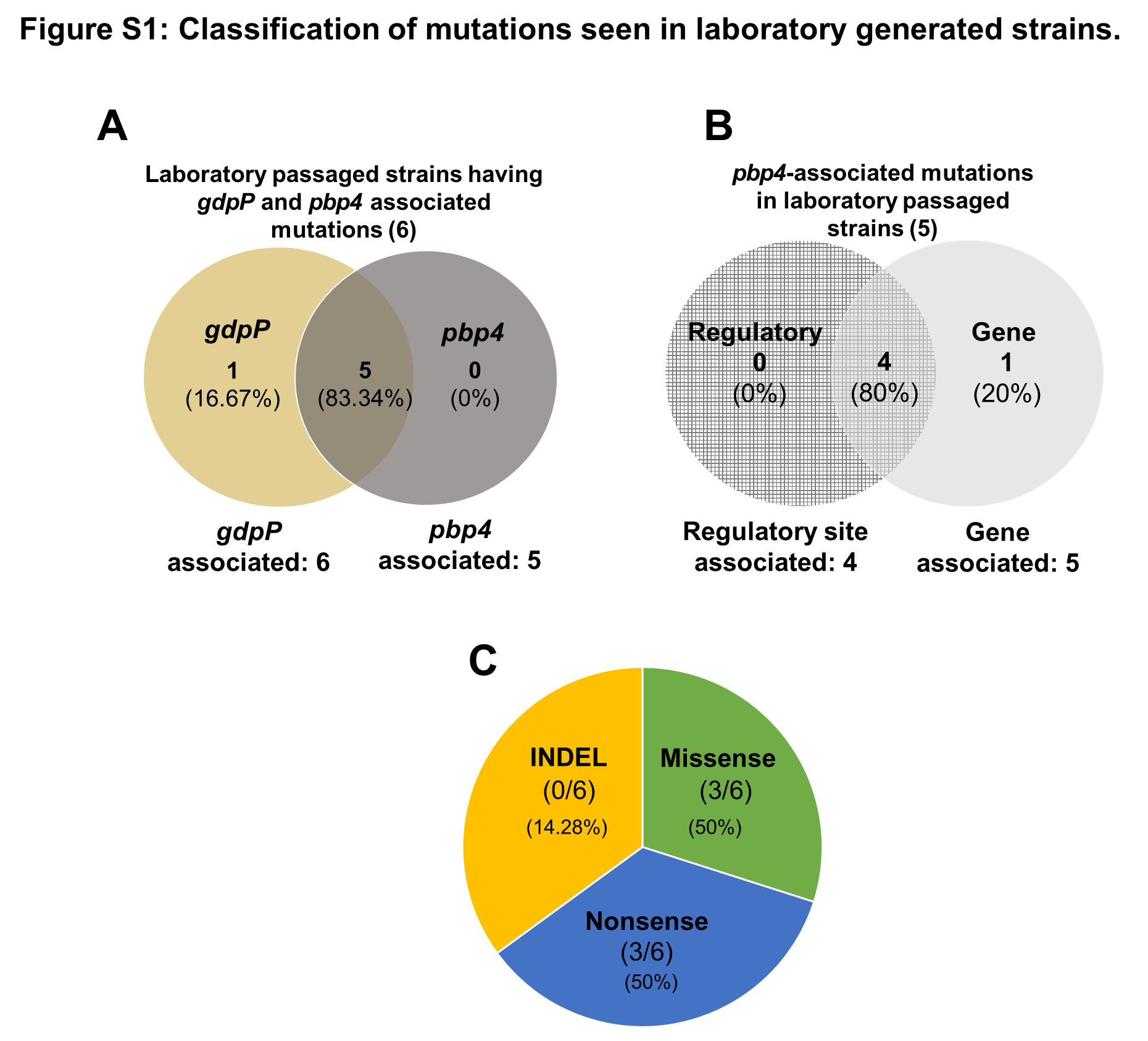
**Figure S1: Classification of mutations seen in laboratory-generated strains.**


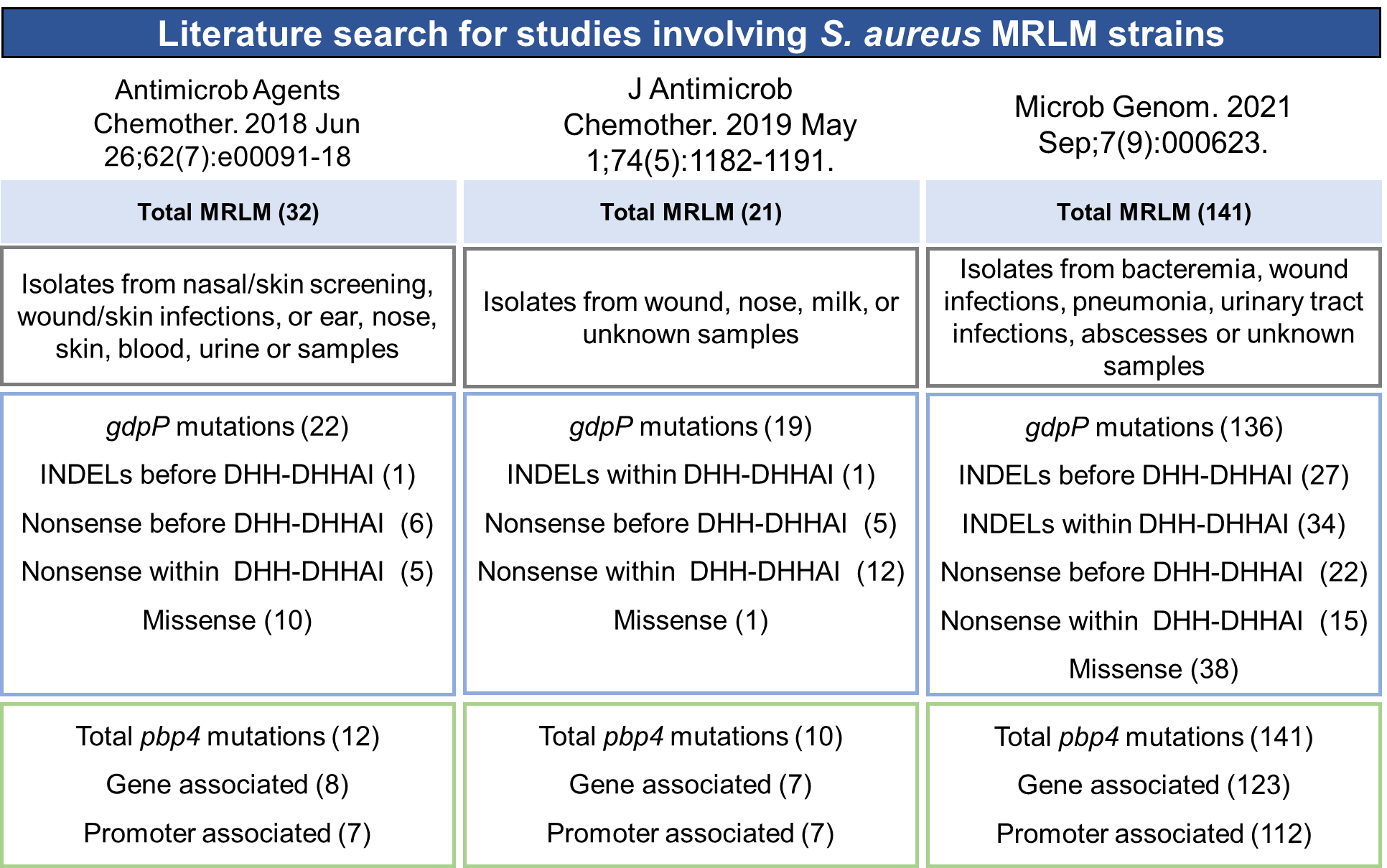


**Figure S2: Schematic representation of classification of MRLM strains from previous studies presented in Figure 1.**


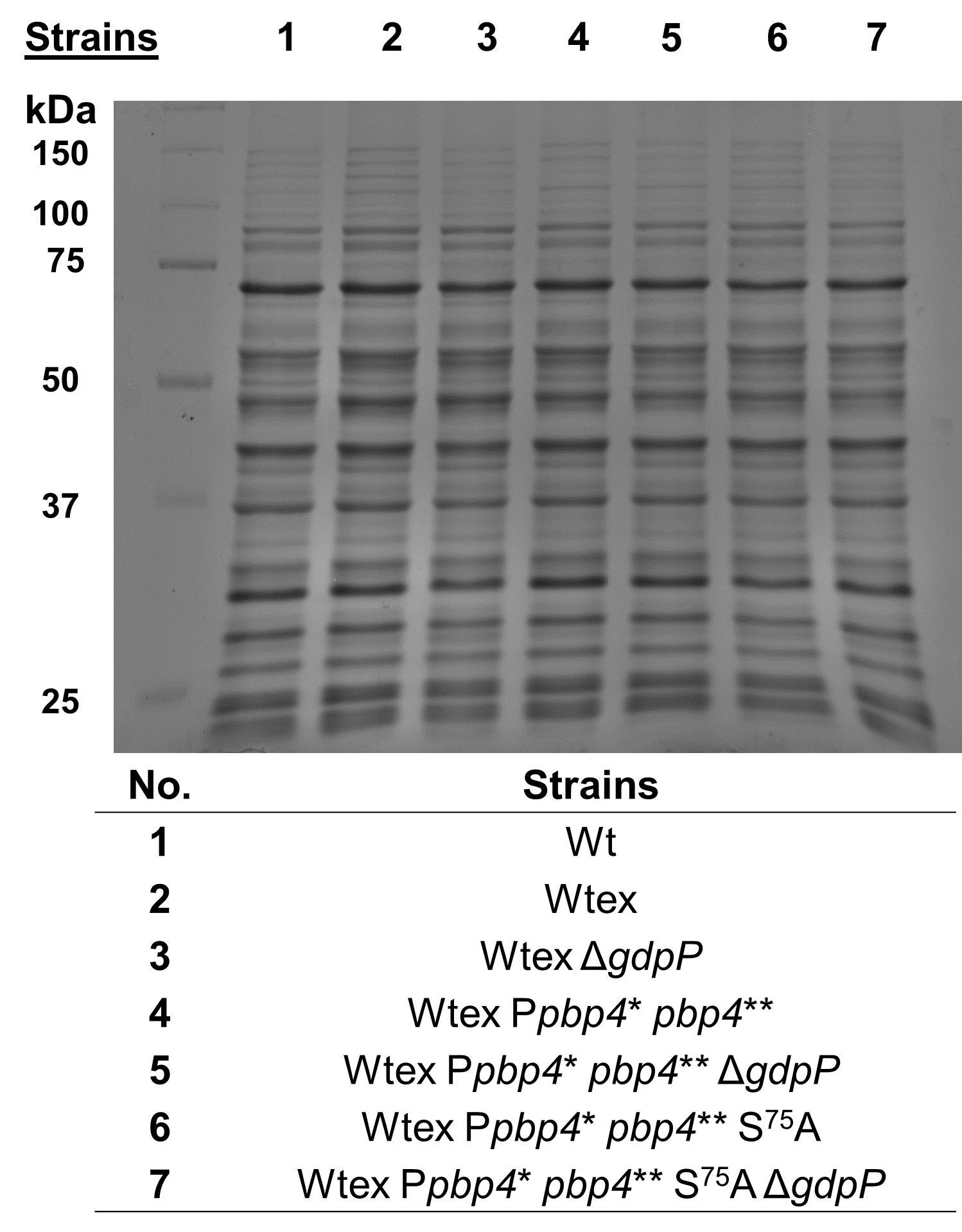


**Figure S3: Loading control for Fig 2D**. Following imaging to detect Bocillin binding to PBPs, the gel presented in **Fig 2D** was stained with Coomassie Brilliant Blue R-250, destained (40% methanol, 10% acetic acid) and imaged using the Azure 600 imager as a loading control.


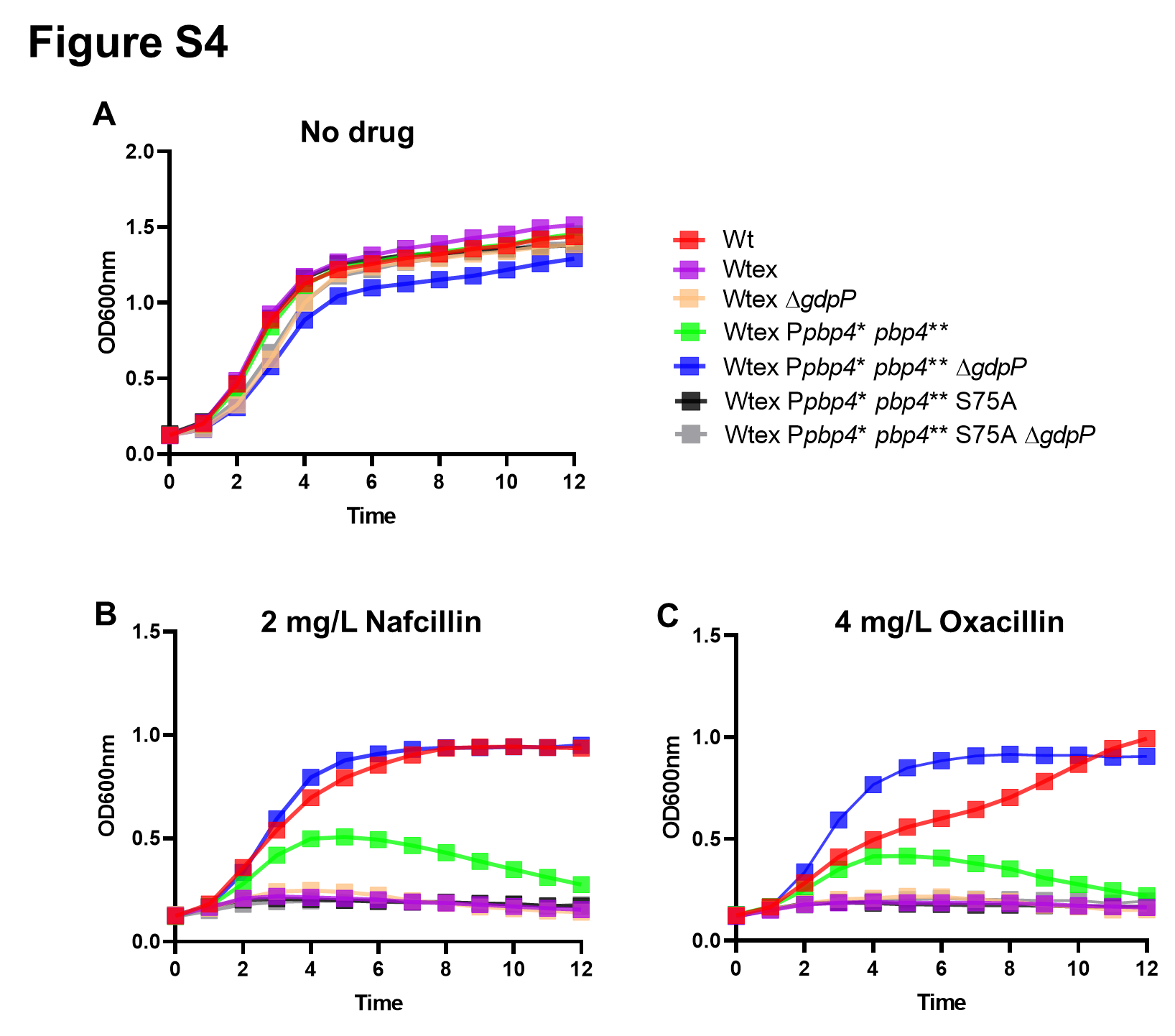


**Figure S4: Growth assay for strains to determine NGB-resistance over a period of time (A)** Without NGBs **(B)** With 2 mg/L Nafcillin **(C)** With 4 mg/L Oxacillin. Deletion of *gdpP* resulted in a growth defect when cultured without NGBs. In presence of NGBs, only Wt and Wtex P*pbp4** *pbp4*** Δ*gdpP* survived.


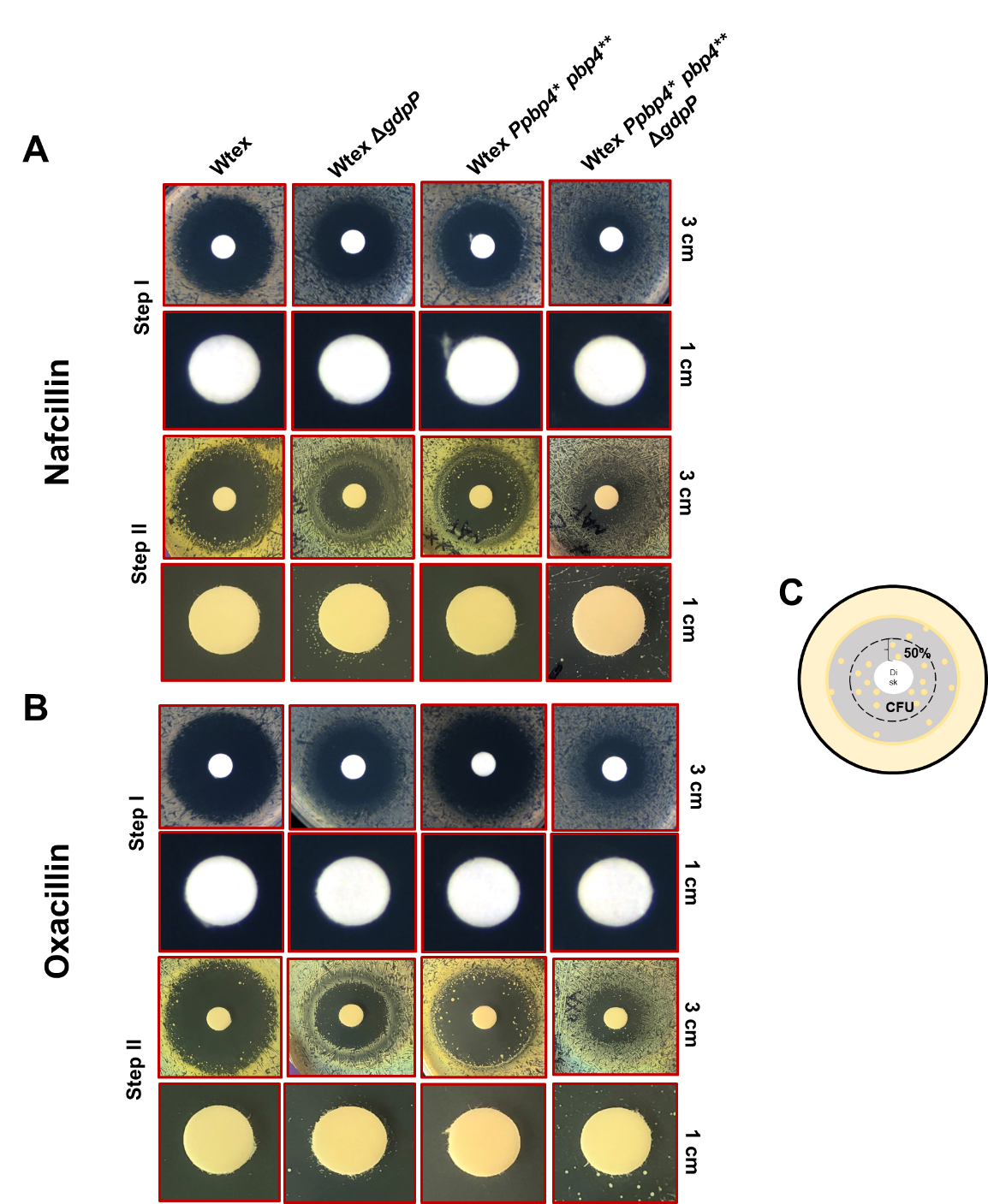


**Figure S5: Effect of the deletion of *gdpP* on antibiotic tolerance by TD test.** Elaborated results of TD-test analysis represented in **Fig 6**. Images shown for step 1 were taken after treatment with **(A)** Nafcillin and **(B)** Oxacillin for 18 hours. Images for step 2 were taken 2 days after treatment with glucose disks. The 2^nd^ row is a magnified image of the disk from the 1^st^ row for each step. While there were no colonies present after glucose exposure for Wtex and Wtex P*pbp4** *pbp4*** strains, they did appear for the strains that contained Δ*gdpP*. Step I shows the inhibition zone on the first day after the bacterial lawn was cultured with a 6 mm disk containing 1 µg antibiotics. There is a clear inhibition zone surrounding the disk. Step II shows the tolerant colonies inside the inhibition zone after replacing the antibiotics disk with a 4 mg glucose disk and culturing for another 2 days. **(C)** The number of tolerant colonies were measured in the inner 50% inhibition zone as demonstarted.


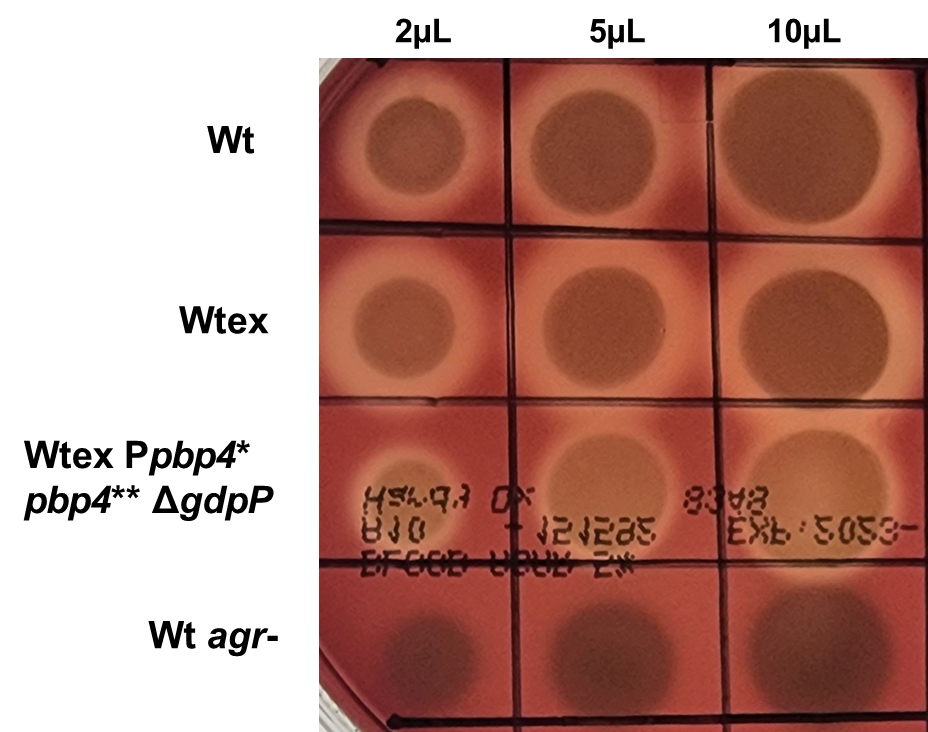


**Figure S6: Hemolysis assay.** Overnight cultures of bacterial strains were spotted onto TSA-blood plates (5% sheep blood) in increasing volumes (2 µL, 5 µL, 10 µL) as indicated, and were incubated at 37°C overnight, following which the plate was stored to 4°C before recording the hemolysis pattern. An *agr* mutant with attenuated hemolysis (Wt agr-) was used as a control.


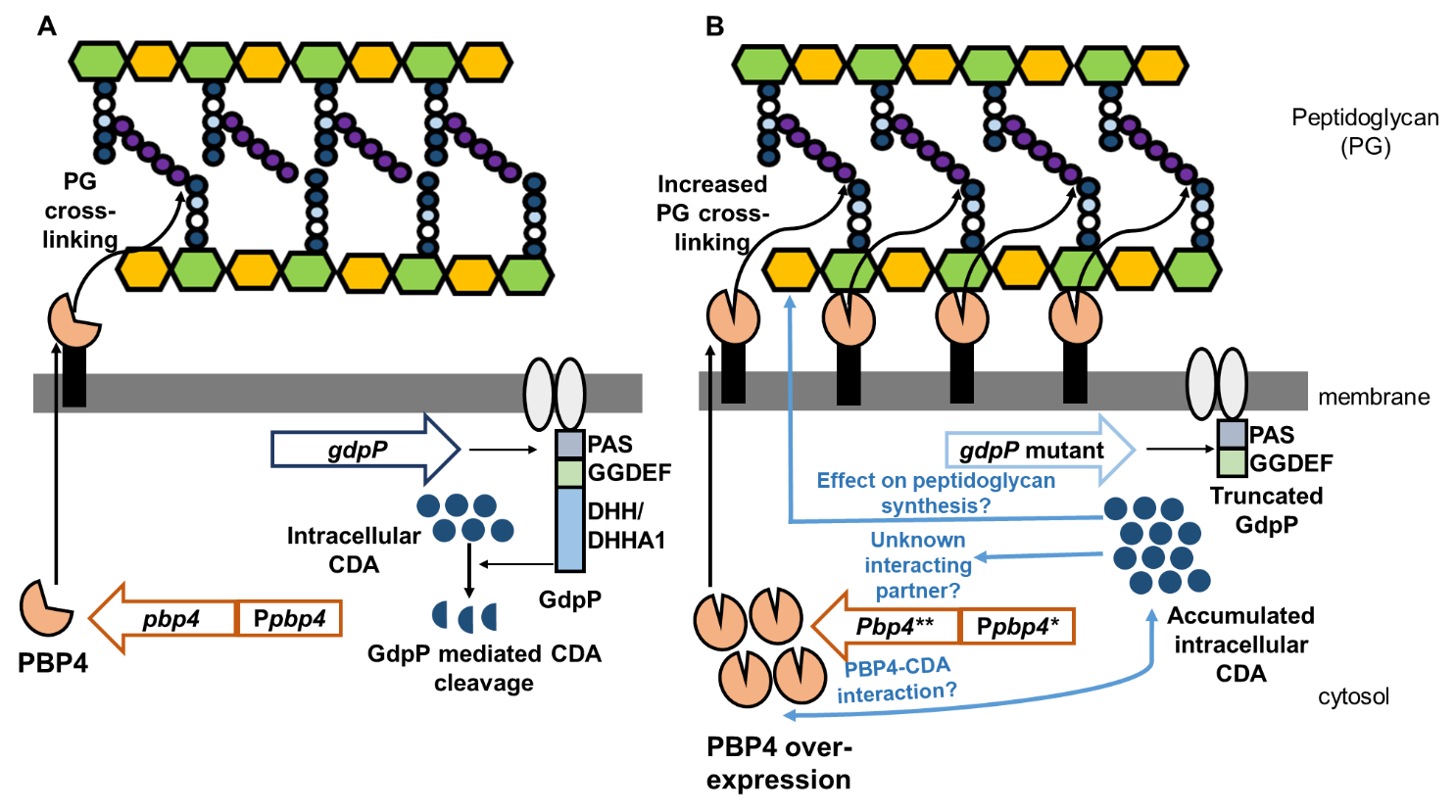


**Fig S7. Schematic representation of the potential mechanisms in which PBP4 and CDA mediate NGB resistance due to alterations in *pbp4* and *gdpP*.** (A) Normal regulation and functioning of PBP4 leads to it first being membrane-bound, following which it performs peptidoglycan cross-linking. Functional GdpP leads to cleavage of CDA, thus maintaining cellular homeostasis. (B) Regulatory-site and missense mutations associated with PBP4 leads to over-expression of a structurally altered protein, that leads to increased peptidoglycan cross-linking. A truncated GdpP, due to mutations associated with the DHH/DHHA1 domain is unable to cleave CDA, causing it to accumulate within the cell. In presene of NGBs, this CDA could either interact directly or indirectly with PBP4 to alter its localization or stability, or could play a peptidoglycan synthesis. Finally, CDA could also have an unindentified interacting partner that plays a role in mediating synergistic NGB resistance.

**References**

1. Chan LC, Gilbert A, Basuino L, da Costa TM, Hamilton SM, Dos Santos KR, Chambers HF, Chatterjee SS. 2016. PBP 4 Mediates High-Level Resistance to New-Generation Cephalosporins in Staphylococcus aureus. Antimicrob Agents Chemother 60:3934-41.

2. Poon R, Basuino L, Satishkumar N, Chatterjee A, Mukkayyan N, Buggeln E, Huang L, Nair V, Argudín MA, Datta SK, Chambers HF, Chatterjee SS. 2022. Loss of GdpP Function in Staphylococcus aureus Leads to &#x3b2;-Lactam Tolerance and Enhanced Evolution of β-Lactam Resistance. Antimicrobial Agents and Chemotherapy 66:e01431-21.

3. Basuino L, Jousselin A, Alexander JAN, Strynadka NCJ, Pinho MG, Chambers HF, Chatterjee SS. 2018. PBP4 activity and its overexpression are necessary for PBP4-mediated high-level β-lactam resistance. J Antimicrob Chemother 73:1177-1180.
